# Effect of psychomotor rehabilitation on rehabilitation exercise in elderly patients with mental disorders and its impact on cognitive function and risk incidence

**DOI:** 10.1097/MD.0000000000047611

**Published:** 2026-03-13

**Authors:** Tingting Jiang, Haiyun Li, Liang Ming, Dandan Mao

**Affiliations:** a Psychiatry Department, Wuhu Hospital (The Fourth People’s Hospital of Wuhu), Beijing Anding Hospital Affiliated to Capital Medical University, Wuhu City, Anhui Province, China.

**Keywords:** cognitive function, elderly mental disorders, psychomotor rehabilitation, rehabilitation exercise

## Abstract

This study investigates the role of psychomotor rehabilitation in the rehabilitation exercise of elderly patients with mental disorders. Eighty-six elderly patients with mental disorders admitted to our hospital from May 2023 to May 2024 were selected as study subjects and randomly divided into 2 groups. The observation group (n = 41) received psychomotor rehabilitation, while the control group (n = 45) underwent conventional rehabilitation exercise. Both groups were continuously intervened for 10 weeks. The Montreal Cognitive Assessment (MoCA), internalized stigma of mental illness scale (a student evaluation version), social disability screening schedule, dysfunction assessment scale, and incidence of risk events were compared between the groups. Psychomotor rehabilitation demonstrates ideal effects, effectively enhancing cognitive function in elderly patients with mental disorders, reducing internalized stigma, improving dysfunctional conditions, alleviating social function deficits, and lowering the occurrence of risk events. It is safe and worthy of clinical application. Before intervention, there was no statistically significant difference in MoCA scores between the groups (*P* >.05). After intervention, the scores in attention, language ability, abstract thinking, and short-term memory improved in both groups, with the observation group showing significantly higher scores than the control group (*P* <.05). Before intervention, there was no significant difference in internalized stigma of mental illness scores between the groups (*P* >.05). After intervention, the scores for stigma resistance, alienation, social withdrawal, stereotype endorsement, discrimination, and total score decreased in both groups, with the observation group showing significantly lower scores than the control group (*P* <.05). Before intervention, there were no significant differences in social disability screening schedule and dysfunction assessment scale scores between groups (*P* >.05). After intervention, both scores decreased in both groups, with the observation group significantly lower than the control group (*P* <.05). The incidence of risk events (mania, aggression, impulsivity, falls, etc) in the observation group was significantly lower than that in the control group (*P* <.05).

## 1. Introduction

Elderly mental disorders are common psychiatric conditions, primarily referring to various types of mental illnesses that occur in individuals aged 60 years and older, such as cognitive impairment, depression, anxiety disorders, and schizophrenia.^[1]^ According to relevant statistics,^[2]^ approximately 14% of the global population aged 60 and above suffer from mental disorders, with depression and anxiety being the most prevalent. Currently, in addition to pharmacological treatments, clinical management of elderly mental disorders should involve multidimensional interventions to improve patients’ quality of life and prognosis.

Rehabilitation exercise plays a positive role in such patients. From a physiological perspective, it can strengthen physical fitness, promote blood circulation, and enhance immune function. From a psychological perspective, it can stimulate endorphin secretion, improve mood, alleviate anxiety and depressive symptoms, and boost treatment confidence.^[3]^ However, traditional rehabilitation exercises often have limited effects due to their lack of specificity and insufficient focus on comprehensive intervention targeting both brain and body functions.

Psychomotor rehabilitation, on the other hand, is a holistic mind–body therapeutic process that uses physical activity as a medium to awaken psychological states and reconstruct neural functions. This therapy has developed into an independent scientific system abroad, though its development in China began relatively late. Clinical observations on patients with dementia have shown that psychomotor rehabilitation interventions based on its theoretical framework can improve certain aspects of cognitive function and daily living abilities, while also alleviating depressive symptoms.^[4]^ Other studies have found that applying psychomotor rehabilitation to elderly patients with schizophrenia significantly enhances patient satisfaction, medication adherence, and quality of life.^[5]^

However, current clinical research on psychomotor rehabilitation for elderly patients with mental disorders remains limited. Therefore, this study aims to further analyze and explore the effects of psychomotor rehabilitation in the rehabilitation exercise of elderly patients with mental disorders, in order to provide more effective support and guidance for their recovery, facilitating faster overall rehabilitation and reintegration into society.

## 2. Materials and Methods

### 2.1. General information

This study was approved by the Ethics Committee of Wuhu Hospital. A total of 86 elderly patients with mental disorders admitted to our hospital from May 2023 to May 2024 were selected as research subjects (Approval No. 2023-PSY-016). The patients were randomly divided into 2 groups according to a random number table: the observation group received psychomotor rehabilitation, while the control group underwent conventional rehabilitation exercises. The study’s objectives, procedures, risks, and potential benefits were explained in detail to all participants. Both patients and their families provided informed consent, and the study was approved by the hospital’s ethics committee.

### 2.2. Inclusion criteria

1. Met the diagnostic criteria for mental disorders according to the *Chinese Classification and Diagnostic Criteria of Mental Disorders (CCMD*)^[6]^;
2. Stable condition and vital signs;
3. Age ≥ 60 years;
4. Disease duration >1 year;
5. Complete clinical data and ability to cooperate with rehabilitation training;
6. No language disorders and capable of normal communication.

### 2.3. Exclusion criteria

1. Severe physical or organic brain diseases;
2. Fractures, trauma, or other conditions that prevent participation in physical activity;
3. Consciousness disorders;
4. Determined by the researchers to be unsuitable for participation;
5. Withdrawal during the study or poor compliance preventing cooperation.

### 2.4. Methods

Control group: Conventional rehabilitation exercise was adopted. Cognitive-behavioral intervention was provided to offer psychological counseling, using simple language to explain the pathogenesis, clinical symptoms, and pharmacological treatment of the disease, while presenting successful cases to strengthen patients’ treatment confidence. According to the patients’ physical conditions and illness status, activities such as calligraphy, painting, rope skipping, and basketball were organized. Patients were encouraged to participate actively, and those who performed well during activities were rewarded. Rehabilitation exercise guidance was provided, instructing patients to wear loose and comfortable clothing, choose soothing music, and perform head and neck relaxation exercises combined with guided meditation. Each movement was performed within the comfort range of the patient, coordinated with breathing and the rhythm of the music. Patients were asked to focus attention and use imagination to picture themselves in nature – feeling the blue sky, breeze, and lake – and to relax their bodies with the music. After each session, active communication was carried out with the patient to ask about their feelings and experiences. Each session lasted 30 minutes, once per day.

Observation group: Under the guidance of the psychomotor rehabilitation team, patients received psychomotor rehabilitation training for 45 minutes per session, 3 times a week, for a total of 10 weeks (30 sessions). The first 10 minutes of each session were for warm-up training, including 5 minutes of slow and brisk walking and 5 minutes of dynamic stretching or fitness exercises, followed by 40 minutes of specialized training. The specific 30-session training program was arranged as follows: Weeks 1 to 2: Psychomotor assessment I: touch and muscle tone dialogue, bamboo pole connection exercises in pairs or groups, balloon volleyball passing, and tennis relay catching exercises. Relaxation therapy: including active relaxation, passive relaxation, direct and indirect relaxation, local and whole-body relaxation, often combined with verbal guidance and breathing to enhance the relaxation effect; relaxation therapy helps achieve emotional calm, relieve muscle tension, and adjust dysfunctional states. Weeks 3 to 4: Fine motor function rehabilitation, including finger-to-finger movement, opening/clenching fists, kneading clay, picking up items with chopsticks, buttoning/unbuttoning, and stringing beads, to improve fine motor ability, coordination, attention, and concentration. Gross motor function rehabilitation was carried out by designing various movement paths to improve postural adjustment ability, large joint control, daily living ability, and safety during position changes. Weeks 5 to 6: Spatial disorder rehabilitation, including static and dynamic training. Static training referred to spatial positioning using self or external objects as references (up, down, front, back, left, right). After mastering basic spatial-temporal concepts, dynamic training was conducted, such as directional walking and dynamic spatial recognition and positioning. (4) Weeks 7 to 8: Body recognition: naming body parts and self-portrait drawing; body perception and representation: experiences with massage balls, small fitness balls, and body-printing activities; spatial perception: directional walking exercises; time perception: rhythmic tapping exercises with long and short beats; coordination rehabilitation: eye-hand coordination, eye-foot coordination, static coordination, and dynamic coordination, such as pushing rods, static standing posture, and targeted sandbag throwing. Both groups underwent continuous intervention for 10 weeks.

### 2.5. Observation indicators

Before and after the intervention, the 2 groups were compared in terms of the Montreal Cognitive Assessment (MoCA), Internalized Stigma of Mental Illness Scale (ISMI), Social Disability Screening Schedule (SDSS), Dysfunctional Attitude Scale (DAS), and incidence of risk events.

(1) **MoCA**,^[7]^ developed by Professor Nasreddine in 2004, covers attention (6 points), language (3 points), abstract thinking (2 points), short-term memory (5 points), naming (3 points), orientation (6 points), and visuospatial/executive function (5 points). It serves as a rapid screening tool for assessing cognitive function, with a total score of 30 points. Higher scores indicate better cognitive ability. Cronbach α coefficient is 0.874, test–retest reliability is 0.841, and content validity is 0.813, indicating good reliability and validity.

(2) **ISMI**^[8]^ includes 5 dimensions: stigma resistance (5 items), alienation (6 items), social withdrawal (6 items), stereotype endorsement (7 items), and discrimination (5 items), with a total of 29 items. It mainly measures the subjective experience of self-stigma among patients. A 4-point Likert scale (1–4 points) is used, with a total score of 116 points. Higher scores indicate stronger self-stigma. Cronbach α coefficient is 0.862, test-retest reliability is 0.921, and content validity is 0.875, indicating good reliability and validity.

(3) **SDSS**,^[9]^ developed in 1988, mainly evaluates the degree of social dysfunction. It includes 10 items, scored on a 2-point Likert scale from “no defect” to “severe defect,” with 0 to 2 points for each item and a total score of 20 points. Higher scores indicate greater social dysfunction. Cronbach α coefficient is 0.893, test-retest reliability is 0.860, and content validity is 0.835, showing good reliability and validity.

(4) **DAS**,^[10]^ developed in 1978, is used to measure maladaptive life attitudes or beliefs. It covers 8 dimensions: compulsiveness, vulnerability, perfectionism, dependency, cognitive philosophy, attraction and rejection, autonomous attitude, and approval-seeking, with a total of 40 items. A 7-point Likert scale (1–7 points) is used, ranging from “strongly disagree” to “strongly agree,” with a total score of 280 points. Higher scores indicate greater dysfunctionality. Cronbach α coefficient is 0.905, test-retest reliability is 0.871, and content validity is 0.846, showing good reliability and validity.

(5) The incidence of risk events in the 2 groups was recorded, including mania (Bech-Rafaelsen Mania Scale^[11]^ ≥ 6 points, with abnormal verbal or behavioral expression, sudden mood changes, hyperactivity, or excessive energy indicating mania), aggression [modified overt aggression scale^[12]^ ≥ 4 points, with verbal, physical, rude, or destructive behavior indicating aggression], impulsivity (Barratt Impulsiveness Scale [BIS-11]^[13]^ ≥80 points, and impulsive actions without considering consequences indicating impulsivity), and falling from bed (hospital-developed Fall Risk Assessment Form ≥8 points, with rolling or rotating body movements resulting in any part of the body touching the ground indicating a fall).

### 2.6. Statistical analysis

SPSS 25.0 (Chicago) statistical software was used for data analysis. Normality of continuous variables was assessed using the Shapiro–Wilk test, and homogeneity of variance was evaluated using Levene test. Parametric tests were applied when assumptions were met; otherwise, appropriate nonparametric tests were used. Categorical data were expressed as percentages and analyzed using the χ^2^ test. Measurement data conforming to a normal distribution were expressed as mean ± standard deviation (±s) and analyzed using the t-test. Data not conforming to a normal distribution were expressed as median and interquartile range (M [P25, P75]) and analyzed using the nonparametric test. A value of *P*<.05 was considered statistically significant.

## 3. Results

### 3.1. Comparison of clinical data between the 2 groups

There were no statistically significant differences (*P* >.05) between the 2 groups in terms of gender, age, course of disease, body mass index, comorbidities, or disease type. Details are shown in Table 1.

**Table 1:** Comparison of clinical data between the 2 groups.

Group	Male/female	Age [year, (P25, P75)]	Disease duration [year, (P25, P75)]	Body mass index [kg/m^2^, (P25, P75)]	Comorbidities (CHD/hypertension/diabetes)	Disease type (dementia/depression/anxiety disorder)
Observation (n = 41)	18/23	72.03 ± 8.69	5.00 (4.00, 5.00)	22.83 (21.90, 23.40)	15/11/18	16/14/11
Control (n = 45)	20/25	71.54 ± 8.13	5.00 (4.00, 5.50)	23.55 (21.30, 25.10)	17/15/23	13/17/15
χ^2^/t/Z	0.003	0.27	-0.786	-1.094	0.13	1.032
*P*	.96	.788	.432	.274	.937	.597

CHD = coronary heart disease.

### 3.2. Changes in moca scores between the 2 groups

Before the intervention, there was no statistically significant difference in MoCA scores between the groups (*P* >.05). After the intervention, scores across all 7 domains – attention, language function, abstract thinking ability, short-term memory, etc – increased compared to pre-intervention levels within each group. Furthermore, the post-intervention scores in the observation group were significantly higher than those in the control group (*P* <.05). Details are presented in Table 2.

**Table 2:** Changes in MoCA scores between the 2 groups (mean ± SD, points).

Domain	Time point	Observation group (n = 41)	Control group (n = 45)	*t*-value	*P*-value
Attention	Pre-intervention	3.25 ± 1.03	3.17 ± 1.00	0.365	.716
	Post-intervention	5.04 ± 0.68*	4.61 ± 0.75*	2.776	.007
Language	Pre-intervention	1.58 ± 0.22	1.66 ± 0.48	0.977	.331
	Post-intervention	2.41 ± 0.38*	2.19 ± 0.30*	2.993	.004
Abstract thinking	Pre-intervention	0.83 ± 0.26	0.92 ± 0.30	1.48	.143
	Post-intervention	1.41 ± 0.17*	1.16 ± 0.21*	6.031	<.001
Short-term Memory	Pre-intervention	2.69 ± 0.89	2.55 ± 0.75	0.791	.431
	Post-intervention	3.88 ± 1.01*	3.15 ± 0.96*	3.436	.001
Naming	Pre-intervention	1.20 ± 0.36	1.36 ± 0.42	1.888	.063
	Post-intervention	2.38 ± 0.75*	2.05 ± 0.63*	2.216	.029
Orientation	Pre-intervention	3.74 ± 1.11	3.86 ± 1.20	0.48	.632
	Post-intervention	4.92 ± 0.74*	4.51 ± 0.93*	2.248	.027
Visuospatial/executive	Pre-intervention	3.47 ± 1.05	3.69 ± 1.13	0.933	.354
	Post-intervention	4.62 ± 1.40*	4.03 ± 1.26*	2.057	.043

MoCA = Montreal cognitive assessment, SD = standard deviation.

* Indicates a significant difference (*P* <.05) compared to the pre-intervention value within the same group.

### 3.3. Changes in ISMI scores between the 2 groups

Before the intervention, there was no statistically significant difference in ISMI scores between the groups (*P* >.05). After the intervention, scores for stigma resistance, alienation, social withdrawal, stereotype endorsement, discrimination experience, and the total score decreased compared to pre-intervention levels within each group. Furthermore, the post-intervention scores in the observation group were significantly lower than those in the control group (*P* <.05). Details are presented in Table 3.

**Table 3:** Changes in ISMI scores between the 2 groups (mean ± SD, points).

Domain	Time point	Observation group (n = 41)	Control group (n = 45)	*t*-value	*P*-value
Stigma resistance	Pre-intervention	16.20 ± 2.25	15.87 ± 2.09	0.705	.483
	Post-intervention	10.45 ± 1.12*	12.36 ± 1.48*	6.698	<.001
Alienation	Pre-intervention	19.37 ± 2.63	18.95 ± 2.31	0.788	.433
	Post-intervention	13.40 ± 1.46*	15.10 ± 2.03*	4.42	<.001
Social withdrawal	Pre-intervention	20.11 ± 2.76	19.83 ± 2.61	0.483	.63
	Post-intervention	14.73 ± 1.90*	16.22 ± 2.14*	3.401	.001
Stereotype endorsement	Pre-intervention	21.46 ± 2.53	21.73 ± 2.24	0.525	.601
	Post-intervention	15.38 ± 1.12*	16.79 ± 1.59*	4.711	<.001
Discrimination experience	Pre-intervention	15.30 ± 2.51	15.89 ± 2.29	1.14	.258
	Post-intervention	10.21 ± 1.43*	11.76 ± 1.92*	4.212	<.001
Total score	Pre-intervention	92.45 ± 5.36	92.07 ± 5.81	0.314	.754
	Post-intervention	64.17 ± 3.39*	72.25 ± 4.05*	9.979	<.001

ISMI = internalized stigma of mental illness, SD = standard deviation.

* Indicates a significant difference (*P* <.05) compared to the pre-intervention value within the same group.

### 3.4. Changes in SDSS and DAS scores between the 2 groups

Before the intervention, there were no statistically significant differences in SDSS and DAS scores between the groups (*P* >.05). After the intervention, both scores decreased significantly within each group compared to their pre-intervention levels. Furthermore, the post-intervention scores in the observation group were significantly lower than those in the control group (*P* <.05). Details are presented in Table 4.

**Table 4:** Changes in SDSS and DAS scores between the 2 groups (mean ± SD, points).

Group	SDSS		DAS	
	Pre-intervention	Post-intervention	Pre-intervention	Post-intervention
Observation (n = 41)	12.47 ± 3.15	6.95 ± 2.01*	137.18 ± 15.25	105.14 ± 11.39*
Control (n = 45)	12.81 ± 3.27	8.23 ± 2.64*	136.92 ± 15.76	118.36 ± 12.52*
*t*	0.49	2.511	0.078	5.105
*P*	.625	.014	.938	<.001

DAS = dysfunctional attitude scale, SD = standard deviation, SDSS = social disability screening schedule.

Compared with pre-intervention.

* *P* <.05.

### 3.5. Comparison of risk incidence between the 2 groups

The overall risk incidence (including mania, aggression, impulsivity, falling from bed, etc) in the observation group was significantly lower than that in the control group (*P* <.05). Details are shown in Table 5.

**Table 5:** Comparison of risk incidence between the 2 groups (n [%]).

Group	Mania	Aggression	Impulsivity	Falling from bed	Total incidence
Observation (n = 41)	2 (4.88)	1 (2.44)	3 (7.32)	1 (2.44)	7 (17.07)
Control (n = 45)	5 (11.11)	4 (8.89)	6 (13.33)	3 (6.67)	18 (40.00)
χ^2^	–	–	–	–	5.657
*P*	–	–	–	–	.017

## 4. Discussion

Patients with geriatric mental disorders typically present with complex conditions, a tendency for recurrence, and significant treatment challenges. These factors not only severely impact the patients’ physical and mental health but also place substantial time and energy burdens on their families. While most patients can achieve symptom stabilization through medication, adjunctive rehabilitation exercises can contribute to comprehensive social recovery. Consequently, selecting appropriate rehabilitation methods has become a key focus of clinical research.

Due to the presence of cognitive abnormalities in elderly patients with mental disorders – manifested primarily in attention, memory, thinking speed, and comprehension – their daily living and social functioning are directly affected. Studies have found that implementing psychomotor rehabilitation in patients with stable schizophrenia not only alleviates negative symptoms but also enhances their cognitive function levels.^[14]^ The results of this study, which showed no statistically significant difference in MoCA scores between the groups before intervention (*P* >.05), but significant increases in all 7 domains – attention, language function, abstract thinking ability, short-term memory, etc – after intervention, with the observation group demonstrating higher scores than the control group (*P* <.05), further confirm that psychomotor rehabilitation can improve patients’ cognitive abilities. This can be attributed to the specific motor exercises in psychomotor rehabilitation, which help patients gradually improve their focus and reduce distractibility. Additionally, these exercises positively influence thinking and judgment abilities. Techniques such as passive relaxation exercises train the brain’s cognitive flexibility and logical reasoning, enabling patients to make more rational choices in complex situations.^[15,16]^ Lastly, given that elderly patients with mental disorders often experience memory impairment, visual, olfactory, gustatory, and tactile exercises can enhance their ability to store and retrieve information, meeting the memory demands of daily life.

The results of this study showed no statistically significant differences in ISMI, SDSS, and DAS scores between the groups before the intervention (*P* >.05). After the intervention, scores improved in both groups compared to pre-intervention levels, with a significantly greater degree of improvement in the observation group (*P* <.05). These findings are consistent with previous clinical research, which demonstrated that psychomotor rehabilitation in patients with schizophrenia improved their life skills and social abilities, and significantly enhanced their social functioning.^[17]^ This is primarily because elderly patients with mental disorders often experience social impairments, difficulty establishing good interpersonal relationships, and are prone to dysfunctional manifestations such as impulsive and aggressive behaviors. Therefore, psychomotor rehabilitation can help patients gain emotional support, boost self-confidence and social skills, reduce feelings of loneliness and social isolation, while also aiding in emotional regulation and behavioral control. This leads to a reduction in impulsive and aggressive incidents, helps lower the incidence of risks, prevents relapse, and alleviates the burden on families and society.^[18,19]^ Another study pointed out^[20]^ that stigma can significantly lower the self-esteem of elderly patients with mental disorders, causing them to avoid interpersonal interactions and greatly impacting their social functioning. Based on this, psychomotor rehabilitation helps patients better understand their physical and mental states. Through participation in rehabilitation activities, patients can gradually recognize their own strengths and weaknesses, thereby enhancing self-awareness. Furthermore, psychomotor rehabilitation places greater emphasis on cultivating psychological resilience in patients – that is, the ability to adapt and recover in the face of difficulties and challenges – enabling them to adopt positive coping behaviors, achieve emotional stability, and simultaneously reduce stigma. Consequently, psychomotor rehabilitation can positively impact the social functioning, stigma, and dysfunctional attitudes of these patients. Through intervention, patients can gradually improve their social skills, reduce stigma, avoid dysfunctional manifestations, and thereby enhance their quality of life and social adaptability. It should also be noted that the reduction in risk events observed in the psychomotor rehabilitation group may not be solely attributable to the intervention itself. Enhanced nursing supervision, increased staff attention, and improved safety management during structured rehabilitation sessions may have contributed to the observed outcomes. Nevertheless, psychomotor rehabilitation may indirectly reduce risk events by improving emotional regulation, motor coordination, and self-control.

A previous study implementing psychomotor rehabilitation for fall risk intervention in elderly patients found significant improvements in their life skills and knowledge, attitudes, and practices regarding fall prevention.^[21]^ This conclusion aligns with the results of the present study, which showed that the incidence of risks such as mania, aggression, impulsivity, and falling from bed was significantly lower in the observation group than in the control group (*P* <.05). This indicates that psychomotor rehabilitation has a positive mitigating effect on risk incidence. This is because techniques like body scanning in psychomotor rehabilitation promote physical relaxation, help patients regulate their emotions, and prevent manic states. Simultaneously, facilitating communication among patients improves their communication skills and effectively enhances self-control能力, reducing the risk of aggressive and impulsive behaviors. Additionally, the entire psychomotor rehabilitation program focuses on functional training of the body, which can improve balance and coordination, effectively preventing risk incidents such as falls due to physical instability.^[22,23]^ Based on this, psychomotor rehabilitation is safe, can reduce the incidence of risks, and provides a safeguard for patient prognosis.

However, this study still has several limitations. Due to the complex pathological mechanisms of geriatric mental disorders and significant individual differences among patients, responses and outcomes during rehabilitation can vary, posing challenges to the generalizability and applicability of the findings. Moreover, the duration for assessing the effectiveness of psychomotor rehabilitation in this study was relatively short, and long-term follow-up was not conducted, making it difficult to accurately evaluate the sustained rehabilitation effects. Future clinical research should delve deeper into the individual differences among elderly patients with mental disorders to develop more tailored protocols. Extending the follow-up period to observe long-term effects would allow for a more accurate assessment of the rehabilitation outcomes and provide better guidance for meeting the rehabilitation needs of this population. This was a single-center study with a relatively small sample size, which may introduce selection bias and limit the generalizability of the findings. Second, although assessor blinding and standardized training were applied, the use of subjective assessment scales may still carry a risk of information bias.

In summary, for elderly patients with mental disorders, psychomotor rehabilitation demonstrates significant effectiveness. It can effectively enhance cognitive function, reduce stigma, improve dysfunctional attitudes and social functioning deficits, and decrease the incidence of risk events, with a high safety profile. It is worthy of clinical promotion and application.

## Author contributions

**Conceptualization:** Tingting Jiang, Haiyun Li, Liang Ming, Dandan Mao.

**Data curation:** Tingting Jiang, Haiyun Li, Liang Ming, Dandan Mao.

**Formal analysis:** Tingting Jiang, Haiyun Li, Liang Ming, Dandan Mao.

**Funding acquisition:** Tingting Jiang, Haiyun Li.

**Investigation:** Tingting Jiang, Haiyun Li.

**Writing – original draft:** Tingting Jiang, Haiyun Li, Dandan Mao.

**Writing – review & editing:** Tingting Jiang, Haiyun Li, Dandan Mao.
